# Infective Endocarditis After Aortic Valve Replacement: A Systematic Review and Meta-Analysis of Risks and Realities in Transcatheter Versus Surgical Repair

**DOI:** 10.7759/cureus.86573

**Published:** 2025-06-23

**Authors:** Moiuz Chaudhri, Ahmed D Al Mahrizi, Muhammad F Ali, Mohammad Hammad, Raviv Markovitz, Jodie Borgmann, Harman Gill, Frederick Acquah, Christian Kaunzinger, Muhammad R Raza

**Affiliations:** 1 Internal Medicine, Ocean University Medical Center, Brick, USA; 2 Education, Futures Forward Research Institute, Toms River, USA; 3 Medicine &amp; Surgery, University of Malta, Msida, MLT; 4 Internal Medicine, Jinnah Postgraduate Medical Center, Karachi, PAK; 5 Internal Medicine, University of Missouri Kansas City School of Medicine, Kansas City, USA; 6 Medicine, Rowan-Virtua School of Osteopathic Medicine, Stratford, USA; 7 Internal Medicine, Jersey Shore University Medical Center, Neptune City, USA; 8 Cardiology, Ocean University Medical Center, Brick, USA

**Keywords:** aortic valve surgery, infective endocarditis, surgical aortic valve replacement, transcatheter aortic valve replacement, valvular heart disease

## Abstract

Aortic valve replacement is an effective intervention for valvular heart disease. However, it carries a rare but serious risk of infective endocarditis (IE). With the expanding use of transcatheter aortic valve replacement (TAVR) in younger, lower-risk patients, it is critical to evaluate its IE risk compared with that of surgical aortic valve replacement (SAVR). We hypothesized that TAVR and SAVR have comparable IE incidence rates but distinct risk profiles influenced by procedural and patient-specific factors.

A total of 17 articles were included in our study. Seventeen studies were included in the qualitative synthesis, and seven studies (118,443 patients) were included in the quantitative analysis. Studies focusing on the incidence of IE after TAVR and SAVR were included. The study was conducted in accordance with Preferred Reporting Items for Systematic Reviews and Meta-Analyses (PRISMA) and was registered.

There was no significant difference in IE risk between TAVR and SAVR (pooled log risk ratio (RR): -0.006, 95% CI (-0.198, 0.185), p = 0.948), although risk estimates varied widely (-1.14 to -1.57), with high heterogeneity (I² = 96%). Major risk factors included valve-in-valve procedures (RR = 2.88), pacemaker implantation (hazard ratio (HR) = 1.91), and chronic kidney disease (HR = 2.08). In-hospital mortality was high (TAVR: 34.4%, SAVR: 31.8%), with over 60% mortality at five years.

TAVR and SAVR have similar rates of IE, but differences in data highlight the need for consistent reporting and personalized risk assessment. Better monitoring, especially for pacemakers or patients with kidney disease, could help improve outcomes. Since prosthetic valve infections have high death rates, guidelines for removing infected TAVR valves may need to be reconsidered.

## Introduction and background

Aortic stenosis is a prevalent cardiovascular disease that affects approximately 40% of individuals over the age of 75 [1]. The clinical manifestations are heterogeneous, ranging from asymptomatic disease to heart failure, angina, and syncope; complications are primarily attributable to decreased cardiac output caused by vascular obstruction [2]. Until recently, surgical aortic valve replacement (SAVR) was the only definitive treatment for patients with severe aortic stenosis. However, in recent years, transcatheter aortic valve replacement (TAVR) has been developed as a less invasive alternative with similar or better outcomes, particularly in high-risk and elderly patient cohorts [3]. Concern about long-term complications has become more pertinent as TAVR has been adopted for younger and lower-risk patients [4]. One of the most serious complications of valve replacement is infective endocarditis (IE), which has a high morbidity and mortality. Before antibiotics, IE was universally fatal, and in modern medical practice, it still has a 30-day mortality rate of up to 30% [5]. Congenital heart defects, bicuspid aortic valve disease, diabetes, immunosuppression, and chronic kidney disease (CKD) greatly increase susceptibility, with an annual incidence of IE in the general population of ∼10 cases per 100,000 people [6].

Although IE after TAVR and SAVR is rare, studies have reported that the incidence of IE after TAVR and SAVR ranges from 0.3 to 2.0 cases per 100 person-years, with no clear superiority of either approach [7]. However, as TAVR use increases, particularly in younger and lower-risk patients, the need to evaluate infection risk, optimal antimicrobial prophylaxis, and early detection strategies is even more pressing [8]. Given that prosthetic valve IE has a poor prognosis, understanding patient-specific risk factors and evidence-based prevention and treatment is important in practice, as are strategies for optimizing our clinical outcomes.

Existing studies have reported conflicting findings and heterogeneity in terms of risk factors and survival. Therefore, a systematic review and meta-analysis was needed to clarify these uncertainties, identify critical risk factors, and guide clinical management strategies for the prevention and treatment of patients with IE.

Thus, this analysis compares the rates of IE after TAVR with those following SAVR, identifies risk factors that may predispose individuals to postprocedural IE, and evaluates clinical presentations, preventative approaches, and treatment modalities. Moreover, this analysis highlights the mediating power of comorbid conditions on IE susceptibility, leading to more precise preprocedural risk stratification and ultimately informing optimal long-term management of affected patients.

## Review

Methods

Search Strategy

The protocol for the study was prospectively registered in PROSPERO (CRD42025632019), and the study was performed according to the Preferred Reporting Items for Systematic Reviews and Meta-Analyses (PRISMA) guidelines [9]. We performed a systematic literature search of four electronic databases, including PubMed, Embase, Scopus, and the Cochrane Library. The last search of the database was conducted in February 2024 without geographic restrictions. Only articles published from 2015 to 2024 were included in the search to focus on contemporary data. Studies incorporating newer-generation TAVR devices are needed. The search strategy included MeSH terms and keywords for IE, aortic valve replacement, and relevant risk factors. The search string for PubMed and Cochrane was (“infective endocarditis” OR “Endocarditis”) AND (“aortic valve replacement” OR “valve replacement” OR “TAVR” OR “TAVI” OR “SAVR”) AND (“risk” OR “incidence” OR “outcomes”). The search string for Scopus was TITLE-ABS-KEY (“infective endocarditis” OR “endocarditis”) AND (“surgical aortic valve replacement” OR “SAVR”) AND (“transcatheter aortic valve replacement” OR “TAVR” OR “TAVI”) AND (“incidence” OR “risk” OR “outcomes”) AND (“comparison” OR “relative risk”) AND (LIMIT-TO (DOCTYPE, “ar”) OR LIMIT-TO (YEAR, “2018-2025”)). The Embase search strategy was as follows: (('infective endocarditis') OR ('endocarditis') AND (('aortic valve replacement') OR ('SAVR') OR ('TAVR') OR ('TAVI')) AND (('risk') OR ('incidence') OR ('outcomes')).

Articles were imported into Zotero (Corporation for Digital Scholarship, Vienna, VA, US) after retrieval for citation management and then uploaded to Rayyan.ai (Rayyan Systems Inc., Cambridge, MA, US) for screening. Duplicate studies were removed through automated and manual verification. Three authors (RM, MC, and MH) screened studies using a stepwise approach, starting with title and abstract screening to determine eligibility and followed by full-text review for final inclusion. Disagreements were resolved by discussion with a fourth author (FA). When full-text access was unavailable, the corresponding authors were contacted and were provided four weeks to respond; a minimum of three attempts were made to obtain the studies. Studies without data available beyond this period were excluded.

Eligibility Criteria

Studies were eligible if their primary data described IE incidence in adult patients (≥18 years) who underwent either TAVR or SAVR, with a follow-up duration of at least six months. Studies included only those peer-reviewed and conference proceedings with full-text availability. Studies were included if adequate data were provided that were amenable to meta-analysis (e.g., incidence rates, risk ratios (RRs), and event rates) and the outcomes were related to IE. Additionally, we included studies that were randomized controlled trials (RCTs), prospective or retrospective cohort studies, and case-control studies. Studies with fewer than 30 patients in each group, single-patient case reports, case series with fewer than 10 patients, studies not providing separate data for TAVR and SAVR, non-English publications without available translations, or abstract-only publications with insufficient information were the exclusion criteria. Studies in patients with congenital heart disease or previous valve surgery not related to TAVR or SAVR were also excluded.

Data Extraction and Outcomes

**Figure 1:**
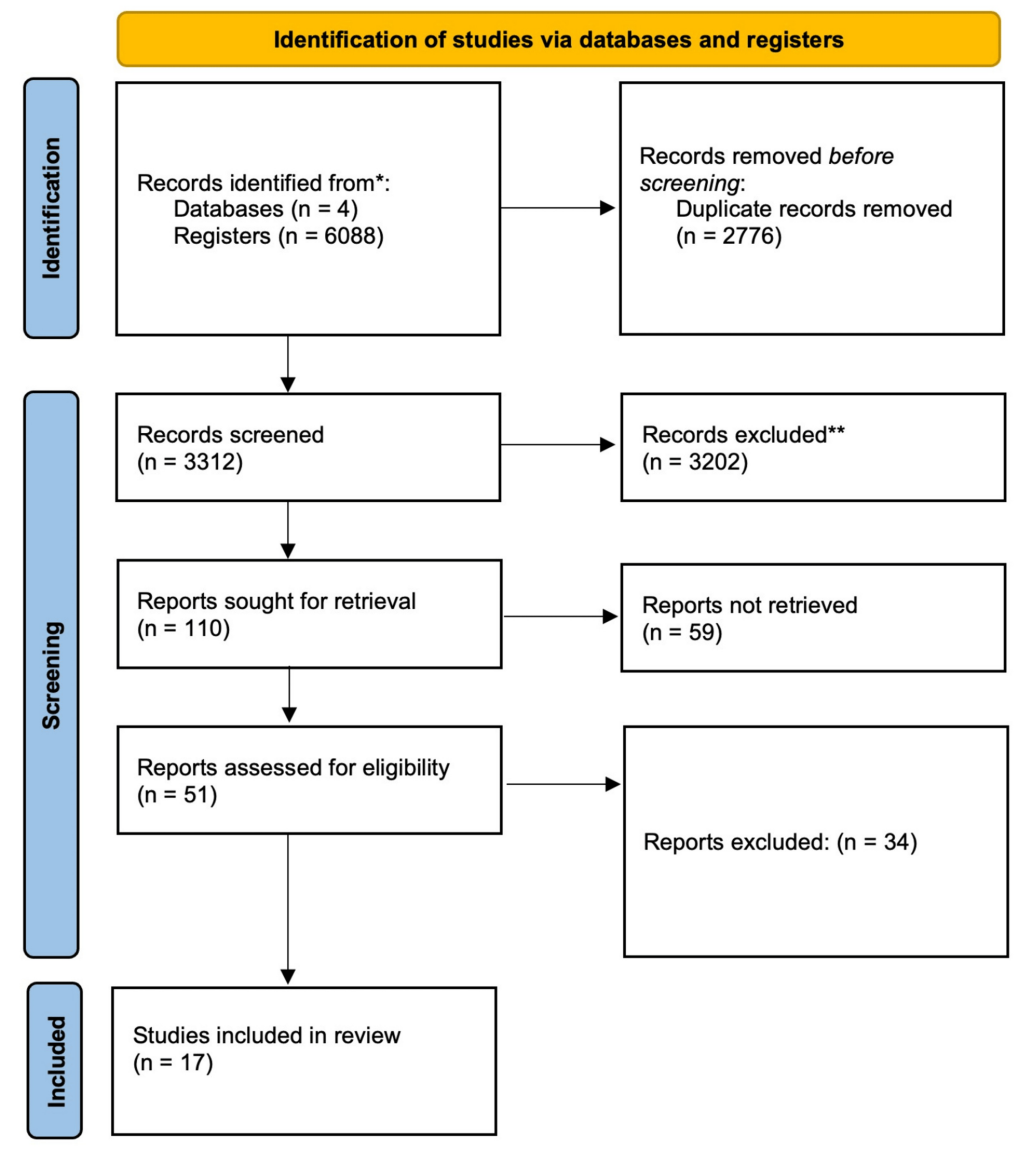
PRISMA flow diagram of the study selection process. PRISMA: Preferred Reporting Items for Systematic Reviews and Meta-Analyses *Embase, Cochrane, PubMed, and Scopus **Did not satisfy eligibility criteria, incorrect study type

Three authors independently extracted the data and verified their accuracy. A total of 6,088 articles were identified through a systematic search. After 2,776 duplicates were excluded, 17 studies were included in the qualitative synthesis, and seven studies (n = 118,443 patients) were included in the quantitative analysis [10-26]. Subgroup analyses were performed on the basis of study design, and publication bias was assessed via funnel plots and Egger’s test. The PRISMA flow diagram shows the study selection process (Figure 1). We extracted variables, including the primary outcomes (which included the incidence of IE in each group and follow-up duration) as well as secondary outcomes, which included patient demographics (age, sex, and comorbidities such as diabetes, CKD, and coronary artery disease), frailty scores, and procedural characteristics. Other extracted variables included the valve type (bioprosthetic or mechanical), valve size, and valve-in-valve status. Data on IE risk factors, including previous IE, immunosuppression, pacemaker or implantable cardioverter-defibrillator (ICD), and concomitant operations, such as coronary artery bypass grafting (CABG), were also collected. Study characteristics (design: RCT, cohort, or case-control analysis; geographic location; year of publication; and sample size) were collected. When available, clinical outcomes related to IE (mortality (in-hospital, 30-day, and long-term), incidence of heart failure, surgical reintervention, and stroke) were obtained (Table 1).

**Table 1:** Baseline characteristics of the included studies. TAVI: transcatheter aortic valve implantation; SAVR: surgical aortic valve replacement; IE: infective endocarditis; AVR: aortic valve replacement; AV: atrioventricular; TAVR: transcatheter aortic valve replacement; PVE: prosthetic valve endocarditis; HR: hazard ratio; nTPV: non-TAVI prosthetic valve; STS: Society of Thoracic Surgeons; THV: transcatheter heart valve

Author	Year	Country	Study design	Sample size (n)	Intervention	Comparator	Mean age (years)	Male (%)	Hypertension (%)	Diabetes mellitus (%)	Hyperlipidemia (%)	Pacemaker implantation (%)	Hospital mortality (%)
Kolte et al. [10]	2018	USA	Nationwide Readmissions Database study	95,383	TAVI	SAVR	81.3 TAVI, 67.0 SAVR (unmatched)	TAVI 52.4%, SAVR 61.4%	TAVI 79.9%, SAVR 73.2%	TAVI 34.6%, SAVR 27.3%	TAVI 59.9%, SAVR 56.5%	10.5% TAVI	15.6% for IE readmission
Dingen et al. [11]	2024	Scandinavia	Multicenter case-control study	847	AVR for IE	AVR for non-infectious disease	IE: 58.5, controls: 68.7	IE 80.0%, controls 59.2%	IE 45.9%, controls 66.3%	IE 14.8%, controls 15.4%	IE 24.7%, controls 50.7%	AV block: 15.9% in IE group	Early mortality IE 7.1%; 6-year mortality IE 40%
Fauchier et al. [12]	2020	France	Cohort	47,553 (TAVI), 60,253 (SAVR)	TAVR	SAVR	75.0 ± 9.5	48.5 (TAVI), 63.5 (SAVR)	84.6 (TAVI), 78.9 (SAVR)	30.9 (TAVI), 30.8 (SAVR)	50 (TAVI), 56.3 (SAVR)	20.5 (TAVI), 6.5 (SAVR)	Mortality higher in TAVI with IE
Shehada et al. [13]	2018	Germany	Prospective single-center study	200	TAVI	SAVR	81 (TAVI) vs. 69 (SAVR)	58 (TAVI) vs. 60 (SAVR)	Higher in TAVI	Higher in TAVI	Higher in TAVI	Not specified	2-year mortality: TAVI 28%, SAVR 16%
Bjursten et al. [14]	2019	Sweden	Nationwide retrospective study	4,336	TAVI	N/A	82	61.1	Not specified	Not specified	Not specified	Not specified	1-year survival after PVE 58%, 5-year survival 29%
Stortecky et al. [15]	2020	Switzerland	Prospective registry (SwissTAVI)	7,203	TAVR	Not specified	Not specified	Male sex associated with higher risk	Not specified	Associated risk factor	Not specified	Associated with higher risk	Mortality HR 6.55 for IE patients
Panagides et al. [16]	2024	International	Registry-based matched cohort study	1,688	TAVR	SAVR	TAVR 80, SAVR 73.8	Not specified	Not specified	More common in TAVR	Not specified	Not specified	1-year mortality ~45% both groups
Strange et al. [17]	2023	Denmark	Nationwide registry study	273 (TAVI), 1,022 (nTPV), 5,376 (native valve IE)	TAVI	nTPV and native valve IE	82 (TAVI), 76 (nTPV), 71 (native valve IE)	66.3 (TAVI), 72.4 (nTPV), 66.6 (native valve IE)	Not specified	16.5 (TAVI), 17.6 (nTPV), 15.5 (native valve IE)	Not specified	Not specified	5-year mortality 75.2%
Fukuhara et al. [18]	2023	USA	Registry study (STS Database analysis)	6,257	TAVR	SAVR	73 (TAVR), 63 (SAVR)	74.5 (TAVR), 79.8 (SAVR)	90.6 (TAVR), 76.6 (SAVR)	44.1 (TAVR), 29.6 (SAVR	Not specified	Higher in TAVR group	13.6% TAVR vs. 10.8% SAVR
Ali et al. [19]	2020	UK	Retrospective cohort study	1,337	TAVI	None	81.3	Not specified	Not specified	Not specified	Not specified	Not specified	In-hospital mortality 38.5%
Ried et al. [20]	2024	Germany	Single-center retrospective cohort	5,716	TAVI	SAVR	80 (TAVI), 67 (SAVR)	60 (TAVI), 83.8 (SAVR)	95 (TAVI), 78.4 (SAVR)	20 (TAVI), 24.3 (SAVR)	Not specified	Not specified	1-year survival TAVI-PVE 90.9% (antibiotics), 33.3% (surgery)
Cahill et al. [21]	2022	UK	National database study	106,157	TAVR	SAVR	Not specified	Not specified	Not specified	Not specified	Not specified	Not specified	Not specified
Calderón-Parra et al. [22]	2023	Spain	Prospective observational cohort with propensity score analysis	633	TAVI	SAVR	83 (TAVI), 69 (SAVR)	55.9 (TAVI), 63.6 (SAVR)	79.5 (TAVI), 69.6 (SAVR)	42.4 (TAVI), 26.8 (SAVR)	Not specified	Not specified	Not specified
Saha et al. [23]	2022	Germany	Multicenter retrospective study	69	Surgery for IE post-TAVR	Not specified	78	69.6	91.3	30.4	47.8	13	In-hospital survival 88.4%, 6-month 68%, 1-year 53%
Kaur et al. [24]	2022	USA	Single-center retrospective cohort	10	TAVR-associated IE (early, intermediate, late)	Not specified	78.1	50	Not specified	Not specified	Not specified	Not specified	Overall mortality 40%
Marin-Cuartas et al. [25]	2024	International registry	International multicenter registry study	372	THV explant for IE	THV explant for bioprosthetic valve dysfunction	74.3	65.8	Not specified	63.7	Not specified	22.5	In-hospital 12.1%, 30-day 16.1%, 1-year 33.8%
Thyregod et al. [26]	2024	Denmark	Randomized controlled trial (NOTION trial)	280	TAVI (CoreValve)	SAVR (bioprosthetic valve)	79.2 (TAVI)/79.0 (SAVR)	53.8 (TAVI)/52.6 (SAVR)	71 (TAVI)/76.3 (SAVR)	17.9 (TAVI)/20.7 (SAVR)	Not specified	Not specified	All-cause mortality 65.5% at 10 years

Risk of Bias Assessment

**Figure 2:**
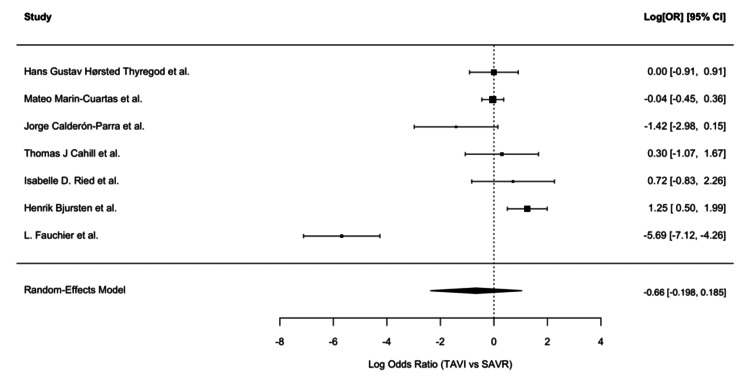
Forest plot with odds ratios of seven studies included in the meta-analysis. Source: Fauchier et al., 2020 [12], Bjursten et al., 2019 [14], Ried et al., 2024 [20], Cahill et al., 2022 [21], Calderón-Parra et al., 2023 [22], Marin-Cuartas et al., 2024 [25], Thyregod et al., 2024 [26] TAVI: transcatheter aortic valve implantation; SAVR: surgical aortic valve replacement; OR: odds ratio

The quality of individual studies was independently evaluated by two reviewers on the basis of the Risk of Bias in Non-randomized Studies of Interventions (ROBINS-I). The risk of bias in this tool was as follows: classified into seven domains: confounding, participant selection, classification of interventions, deviation from intended intervention, missing data, measurement of outcomes, and selection of the reported result (Figure 2). Differences in evaluations were resolved by consensus. The risk of bias summary was plotted visually in a traffic light plot, with the studies individually color-coded as reflecting low (green), moderate (yellow), or high (red) risk of bias.

Data Synthesis and Analysis

The meta-analysis was conducted via RStudio (version 4.1.0) (Posit Software, Boston, MA, US). The pooled log odds ratios (ORs) for dichotomous outcomes (sensitivity and specificity) were estimated with 95% CIs. Heterogeneity was measured with I² and χ², where it was taken to indicate that 50% or more I² represented substantial heterogeneity. Sensitivity analyses were conducted to explore possible sources of heterogeneity. Publication bias was evaluated with funnel plots.

Results

Study Selection

A total of 6,088 articles were identified through a systematic search. After excluding 2,776 duplicates, 17 studies were included in the qualitative synthesis, and seven studies (n = 118,443 patients) were included in the quantitative analysis [10-26].

Study Characteristics

Our sample sizes ranged from 69 to 372 patients across two intervention groups (TAVR and SAVR). We noticed that males represented a majority in most cohorts, with a sex distribution ranging from ~52% to ~70% male. Patients had a range of comorbidities, including CKD, diabetes mellitus, chronic obstructive pulmonary disease (COPD), malignancy, and advanced heart failure. The frailty score has been infrequently reported [10-26].

Meta-Analysis Results

The incidence of IE varies across studies, with TAVR generally associated with a slightly higher or similar rate compared with SAVR. Duration of follow-up ranged from nine months to 10 years, with clinical outcomes such as mortality (in-hospital and long-term), heart failure, stroke, and surgical reintervention reported variably. SAVR patients tended to be younger and healthier. TAVR patients were typically older (e.g., 83 vs. 69 years) and had more comorbidities (CKD, diabetes, and COPD). In-hospital mortality ranged from ~11% to 12%. The long-term mortality rate was high in both groups due to age and comorbidities but was comparable in most studies. One study reported a hazard ratio (HR) for mortality of 1.0 (95% CI: 0.7-1.3), indicating no significant difference. The risk of stroke was variable, but in some cases, it was greater with SAVR (e.g., TAVI 9.7% vs. SAVR 16.4%). Reintervention was generally rare but was more common in IE patients.

**Figure 3:**
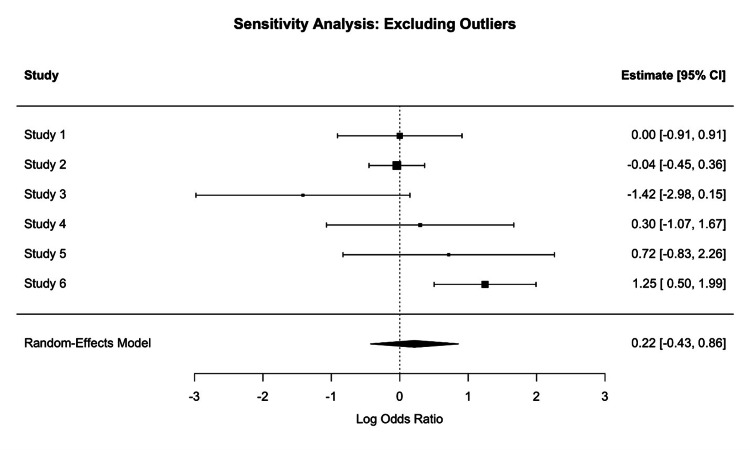
Forest plot showing the sensitivity analysis after excluding outliers. Study 1: Thyregod et al., 2024 [26], Study 2: Marin-Cuartas et al., 2024 [25], Study 3: Calderón-Parra et al., 2023 [22], Study 4: Cahill et al., 2022 [21], Study 5: Ried et al., 2024 [20], Study 6: Bjursten et al., 2019 [14]

**Figure 4:**
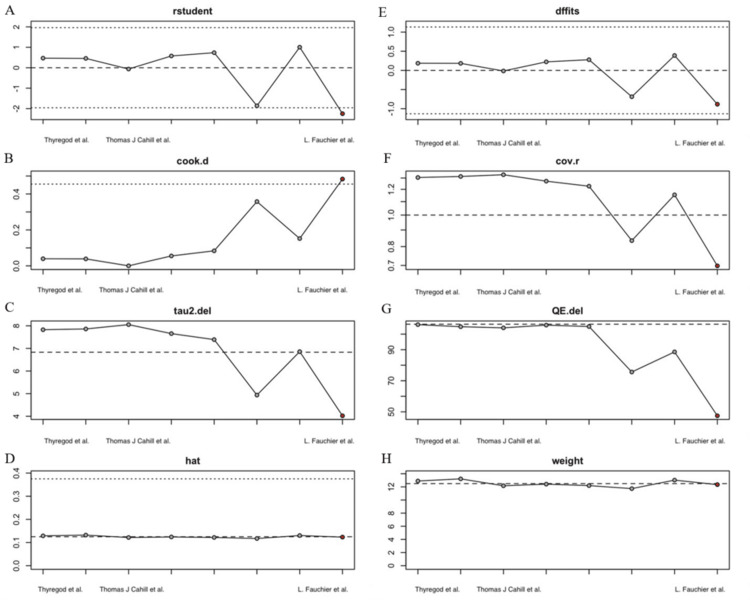
(A-H) Diagnostic and sensitivity analyses of the studies included in the meta-analysis. Source: Fauchier et al., 2020 [12], Cahill et al., 2022 [21], Thyregod et al., 2024 [26]

We reported HRs and ORs, indicating no statistically significant advantage in IE or mortality outcomes for either TAVR or SAVR after adjusting for confounders. Furthermore, the forest plots (Figures 3, 4) show the ORs for IE between TAVR and SAVR across the selected studies. The OR varies across studies, indicating heterogeneity in the results. Only a few studies included Kaplan-Meier survival curves, which showed largely overlapping trends between TAVR and SAVR, indicating comparable long-term survival. Risk factors such as age, comorbid conditions, and valve type were mentioned as potential contributors to IE risk, but consistent predictors across studies were not clearly established.

Meta-regression analysis was considered, focusing on key covariates such as patient age and sex, follow-up duration, study design, and comorbidity burden (e.g., CKD, diabetes, and COPD). Given the variability in clinical settings and sample sizes across the five studies, meta-regression aimed to assess whether these factors are significant and to explore potential sources of the heterogeneity among the included studies revealed a reduced mortality benefit or risk. The difference in IE risk between the two groups was not significant, with a pooled log RR of -0.006 (95% CI: (-0.198, 0.185), p = 0.948). This finding suggests that the choice between TAVR and SAVR does not substantially influence the likelihood of IE development. However, the substantial heterogeneity (I² = 96%) and the wide range of individual study estimates (log RR: -1.14 to -1.57) indicate significant variability across studies, likely due to differences in patient populations, procedural techniques, and follow-up durations or IE diagnostic criteria. Subgroup and sensitivity analyses revealed that the study design may partially explain the observed heterogeneity, although the I² value remained high (94.58%) even after excluding outliers (Figure 3). In particular, the study by Fauchier et al. was noted to have the greatest influence across multiple measures (Figure 4).

**Figure 5:**
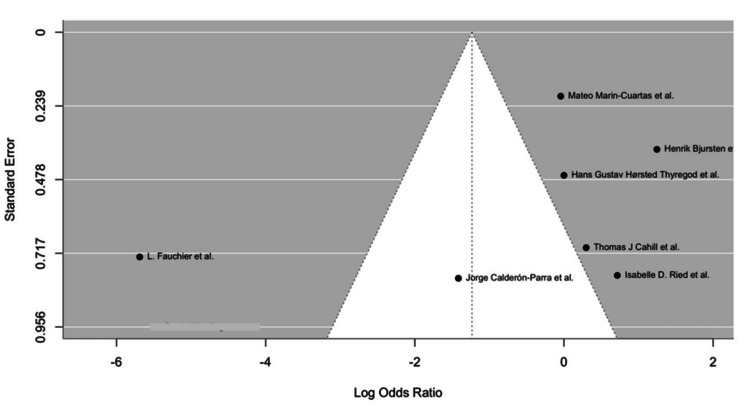
Funnel plot assessing publication bias among the studies included in the meta-analysis. Source: Fauchier et al., 2020 [12], Bjursten et al., 2019 [14], Ried et al., 2024 [20], Cahill et al., 2022 [21], Calderón-Parra et al., 2023 [22], Marin-Cuartas et al., 2024 [25], Thyregod et al., 2024 [26]

Publication bias was assessed via funnel plots (Figure 5). This suggests the potential for some bias in the studies included here.

Quality Assessment

A ROBINS-I assessment was used to assess study bias in the non-randomized studies. In general, the studies showed a moderate to high risk of bias in multiple domains. In the majority of studies, bias from confounding factors was moderate, commonly due to incomplete control for baseline characteristics or concurrent interventions. Selection bias was, on the whole, low as the inclusion criteria were reported in most of the studies. However, bias in the classification of interventions was often rated as serious, particularly due to retrospective definitions of exposure and lack of information regarding the timing of interventions.

In the areas of deviations from intended interventions and missing data, studies were generally classified as moderate (usually indicating unclear reporting of adherence or attrition). Measurement of outcomes was a frequently identified area of concern with a number of studies scoring serious risk in relation to blinding or use of outcome measurement tools with subjective criteria. Lastly, the bias in the selection of the reported result was usually considered low, although selective reporting bias could not be completely excluded in some studies.

Overall, these findings imply that although there were some methodological strengths, this evidence was limited in that it might be affected in its validity of estimated intervention effects (Figure 2).

Discussion

This meta-analysis revealed similar rates of IE between TAVR and SAVR; however, no statistically significant difference between procedures was observed. Different risk factor profiles suggest the contribution of procedural and patient-related factors to susceptibility to infection and clinical outcomes. The incidence of IE after TAVR was between 0.3% and 5.8% (average: 1.1%-1.7% per person-year). The incidence after SAVR ranges from 0.43% to 2.5% (average: 1.4%-2.5% per person-year) [6]. A 2019 large registry dataset from Cahill et al. revealed incidence rates of 3.57 cases per 1,000 person-years for TAVR vs. 4.81 cases per 1,000 person-years for SAVR, with an HR = 1.60 in favor of TAVR [7]. These results indicate a lower rate of IE after TAVR but with substantial heterogeneity between studies, limiting direct comparison.

There are several factors that seem to be associated with an increased risk of developing IE after TAVR and SAVR, and differences in both procedural and patient-specific variables were noted; valve-in-valve TAVR was associated with a 2.88-fold increased risk of IE, possibly a result of residual native valve tissue providing a nidus for bacterial colonization [8]. The need for pacemaker/ICD implantation itself (also not surprising after TAVR) was also associated with a significantly increased risk of IE (49%-83%), possibly caused by lead-induced endocardial injury and bacterial seeding [7]. CKD is another important predictor of IE risk among the TAVR cohort, with a 2.08-fold greater likelihood of having IE, possibly related to immune dysfunction, repeated vascular access, and increased catheter-related infections [14].

However, for SAVRs, the factor that specifically influences the risk of IE is the prosthetic material implanted (such as the prosthetic valve) and the SA for mechanical valves, associated with lower infections due to the lower bacterial adherence than that of bioprosthetic valves [27]. Notably, younger age, usually thought to correlate with a better overall cardiovascular risk profile, is paradoxically associated with an increased incidence of IE in SAVR patients, potentially as a result of a longer duration of bacteremia and greater valve-related stress [28]. Common risk factors associated with TAVR and SAVR are diabetes, male sex, and immunosuppression, suggesting that host factors play a role in infection susceptibility [29].

In addition to incidence and risk factors, IE following TAVR and SAVR is associated with poor clinical outcomes, with one-year mortality rates ranging from 30% to 75% for TAVR-IE and 20% to 65% for SAVR-IE [30]. Although the mortality rates were similar, the reoperation strategies differed significantly between groups. Patients with SAVR-associated IE were more often treated with surgical valve explanation (33%-50%), considering the proportional feasibility of valve debridement and replacement in surgical candidates [31]. In contrast, surgical reintervention was less common with TAVR-IE (15%-20%), presumably owing to technical difficulties with performing transcatheter valve replacement and increased procedural risk in elderly or frail patients [31]. Faced with these limitations, early diagnosis and intensive medical treatment are urgently needed to optimize TAVR-IE management.

Notably, the meta-regression revealed no significant association between sample size and effect size, suggesting that the variability is not solely attributable to differences in the study scale. The included studies, such as the large cohort study by Cahill et al. and the 10-year RCT by Thyregod et al., reported comparable IE incidence rates between TAVR and SAVR (0.36 vs. 0.48 and 0.72 vs. 0.74 per 100 patient-years, respectively) [21,26]. However, other studies, such as Panagides et al. and Fukuhara et al., reported higher but proportionally similar rates (31.8 vs. 38 and 6 vs. 9.4 per 100 patient-years), further underscoring the consistency in relative risk despite the absolute rate disparities [16,18].

The wide variability in absolute incidence rates-ranging from as low as 0.09 in Bjursten et al. to as high as 65.95 in Marin-Cuartas et al., highlights the influence of contextual factors, such as patient risk profiles, particularly those reported by Saha et al. [14,23,25]. Wide variability can also be due to the different types of valves observed in a study by Ried et al. [20]. While the pooled analysis suggests no overall difference in IE risk between TAVR and SAVR, heterogeneity and extreme outliers (e.g., explant registry data) warrant cautious interpretation.

We notice strong associations with pacemaker/ICD implantation (49%-83% increased risk) and valve-in-valve procedures (2.88x higher relative risk). Moreover, CKD increased risk in both groups, but more prominently in the TAVR group (2.08x higher HR). In contrast, SAVR has a lower IE risk with mechanical valves than with bioprosthetics and a paradoxically higher risk in younger patients, possibly reflecting longer exposure times or comorbid conditions.

Some limitations need to be considered when these findings are interpreted. We acknowledge that high interstudy heterogeneity, stemming from differences in follow-up duration, patient selection criteria, and diagnostic definitions of IE, introduces bias and limits the generalizability of pooled estimates of incidence. Moreover, the funnel plot asymmetry also revealed possible publication bias, which means that studies with non-significant results might be overlooked. Finally, long-term data regarding IE risk in next-generation TAVR valves are limited, and continued surveillance in younger and lower-risk populations is warranted.

## Conclusions

This systematic review and meta-analysis demonstrates that the incidence of IE is comparable following TAVR and SAVR. Nonetheless, the clinical implications of IE are influenced by different procedural and patient characteristics. The reported risk may be due to device properties, comorbidities, and procedural complexity. Optimal management of prosthetic valve endocarditis requires close collaboration among interventionalists, surgeons, and infectious disease specialists. With the application of TAVR in younger and lower-risk groups, continued surveillance and long-term investigation are needed to clarify the risk of infection and provide the highest standards of care for our patients.
